# Rethinking arsenal: non-TAL effectors drive *Xanthomonas* virulence in Brassica

**DOI:** 10.1093/plphys/kiag109

**Published:** 2026-02-27

**Authors:** Ritu Singh, Prem Pratap Singh

**Affiliations:** Assistant Features Editor, Plant Physiology, American Society of Plant Biologists; Department of Plant Science, University of California, Davis, CA 95616, United States; Department of Viticulture & Enology, University of California, Davis, CA 95616, United States

Black rot, caused by the bacterial pathogen *Xanthomonas campestris* pathovar *campestris* (*Xcc*), remains one of the most devastating diseases of cruciferous crops worldwide. The pathogen infects nearly all *Brassica*-growing regions and causes severe yield and quality losses (Vicente and Holub 2013). Like many other gram-negative phytopathogenic bacteria, *Xanthomonas* species rely on type III secretion systems to inject virulence proteins directly into host plants, manipulate host immunity, and proliferate within plant tissues (da Silva et al. 2002; Büttner and Bonas 2010). These injected proteins, known as type III effectors, are classified as transcription activator-like effectors (TALEs) and non-TAL effectors (Mondal and Kalaivanan 2023). For decades, the plant pathology community has viewed TALEs as the primary virulence weapon in the *Xanthomonas* arsenal, especially in pathosystems involving rice, pepper, and cassava (Jiang et al. 2008; Li et al. 2015; Liao et al. 2020).

Whether this TALE-focused approach can also be applied to *Brassica* crops remains unresolved. In a recent study published in *Plant Physiology*, Chen and colleagues explore this question with a large geographically diverse collection of *Xcc* strains (Chen et al. 2026). The authors examined 70 strains from China along with representative races from Europe, Australia, and the United States for the presence of *tal* genes encoding TALE effectors. Pathogenicity assays across 5 Brassica accessions revealed substantial variation in disease severity, identifying 6 highly aggressive strains, of which 4 displayed consistently high virulence across multiple host genotypes. Screening for *tal* genes showed that only about 5% of endemic Chinese strains carried detectable *tal* sequences, and overall *tal* prevalence across the 70-strain panel remained low. Notably, several of the broadly virulent strains carried *tal* genes; however, functional analyses demonstrated that deletion of single or multiple *tal* genes did not reduce disease severity, even on susceptible Brassica hosts. These findings indicate that, unlike the well-characterized roles of TALEs in *X. oryzae pv. campestris* and *X. oryzae pv. oryzae* infecting rice, the *tal* genes in Chinese *Xcc* strains do not make a measurable contribution to virulence in the Brassica pathosystem.

If TALEs are not the primary cause of virulence, what enables *Xcc* to cause disease in Brassica? To understand this, the authors performed whole-genome sequencing of 5 representative *Xcc* strains using long-read sequencing on the PacBio Sequel II platform, combined with short-read sequencing on the Illumina NovaSeq platform. Genome analysis revealed a largely conserved but variable repertoire of non-TALE effectors, known as Xanthomonas outer proteins (Xop), across strains. To test the contribution of these effectors to virulence, the authors generated single-gene deletion mutants for 5 representative *xops* (*xopK*, *xopQ*, *xopX-1*, *xopAM*, and *xopN*) and assessed their effects in cabbage (Fig. 1). The results revealed a strong host-dependent function. In the cabbage line G1180, deletion of *xopQ*, *xopX-1*, *xopAM*, or *xopN* increased disease severity and bacterial growth, indicating that these effectors function as avirulence factors that trigger host immune recognition. Conversely, in the cabbage line G87-534, deletion of *xopK* or *xopN* significantly reduced disease severity and bacterial counts, confirming their role as virulence factors in a compatible host. Notably, in G1180, the *xopX-1xopN* double mutant displayed even greater disease severity and bacterial counts than either single mutant, indicating that these 2 effectors jointly contribute to host recognition in this genetic background.

**Figure 1:**
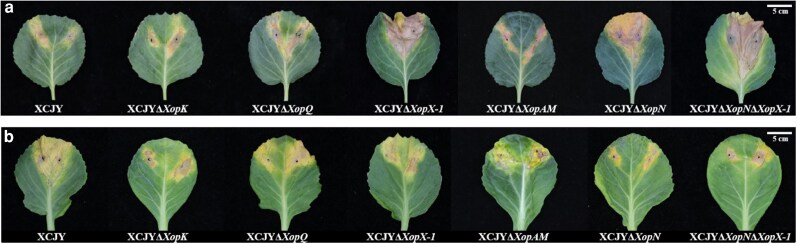
Host-dependent effects of *Xop* effectors on black rot (BR) disease development in cabbage. Representative disease symptoms on cabbage leaves inoculated with the wild-type *Xcc* strain *XCJY* and 6 single *xop* deletion mutants. Leaves from cabbage line G1180 **(a)** and G87-534 **(b)** show BR lesion at 10 days post inoculation. Deletion of individual *xop* genes resulted in contrasting disease outcomes depending on host genotype, with some mutants showing enhanced lesion development in G1180 and reduced disease severity in G87-534. These results highlight host dependent roles of non-TALE *Xop* effectors in *Xcc* virulence.

To elucidate the mechanism by which these Xops manipulate the host immunity, the authors used transient expression assays in *Nicotiana benthamiana* and cabbage. Transient expressions of *XopQ*, *XopX-1*, and *XopN* triggered a hypersensitive response in cabbage, a hallmark of effector-triggered immunity. Quantitative PCR analysis further showed inoculation with 5 of the tested *xop* mutants led to increased expression of *PR1*, *WRKY18*, or *WRKY33* compared with the wild-type strain, suggesting that these effectors normally act to dampen immune signaling. Although direct molecular targets were not identified, the elevated defense gene expression in the deletion lines supports a role for *XopQ*, *XopX-1*, and *XopN* in modulating host immune transcriptional responses. Together, these results demonstrate that Xops operate at the intersection of virulence and recognition. They suppress host immune signaling to promote infection yet can also activate hypersensitive responses depending on the host genotype.

Collectively, this study demonstrates that Chinese *Xcc* strains infecting Brassica rely primarily on non-TAL *Xop* effectors rather than TALEs for pathogenicity. The rarity and functional irrelevance of *tal* genes underscore a fundamental divergence between the *Xcc*-Brassica interaction and other well-studied *Xanthomonas* pathosystems. By providing the first comprehensive type III effector landscape of Chinese *Xcc* strains and revealing strong host-dependent roles for key *Xops*, this work reshapes our understanding of black rot pathogenesis.

The evolutionary implications are notable. Although *tal*-positive strains displayed higher overall virulence, this likely reflects broader genomic features, such as expanded effector repertoires or pathogenicity-associated genomic regions, rather than a direct causal role of TALEs in Brassica infection. Given that *tal* genes were detected in only about 5% of the 70 strains initially screened and that the broader host range of these strains remains incompletely defined, it will be important to determine whether TALE dispensability extends to *Xcc* strains infecting other Brassica species or even alternative hosts. In contrast, *Xops* show clear evidence of functional tuning to specific host genotypes. Future comparative studies examining *tal*-positive and *tal*-negative strains across diverse host backgrounds will clarify whether TALEs represent lineage-specific relics or retain context-dependent roles outside the Brassica pathosystem analyzed here. Identifying host targets of major *Xops*, dissecting effector cooperation and redundancy, and expanding analyses to additional virulence determinants will be essential for understanding *Xcc* adaptation to Brassica and for developing durable resistance strategies in Brassica crops.

## Recent related articles

Üstün and Börnke (2015) showed that the *Xanthomonas campestris* type III effector *XopJ* degrades the proteasome subunit RPT6, suppressing salicylic acid mediated defense signaling in plants.

## Data Availability

No data is generated in this study.
